# European Health Information Portal: a one-stop shop for health information

**DOI:** 10.1093/eurpub/ckad172

**Published:** 2024-07-01

**Authors:** Hanna Tolonen, Miriam Saso, Brigid Unim, Luigi Palmieri, Nienke Schutte, Mariana Peyroteo, Luís Velez Lapão, Claudia Habl, Petronille Bogaert, Population Health Information Research Infrastructure and the Health Information Portal, Claudia Habl, Cara Pries, Richard Pentz, Stefan Mathis-Edenhofer, Andrea Schmidt, Alexander Grabenhofer-Eggerth, Johannes Weiss, Sophie Sagerschnig, Anita Gottlob, Lorenz Dolanski, Alexander Degelsegger-Marquez, Beate Gruber, Katharana Habimana, Petronille Bogaert, Marie Delnord, Nienke Schutte, Kim Vyncke, Tadek Krzywania, Linda Abboud, Miriam Saso, Brecht Devleesschauwer, Barthélémy Moreau de Lizoreux, Pascal Derycke, Pierre Daubresse, Sasha Milbeck, Karin De Ridder, Charles-Andrew Van de Catsyne, Sejla Cilovic Lagarija, Anina Chileva, Jelena Dimnjakovic, Jakov Vukovic, Sarka Dankova, Ondrej Májek, Sigrid Vorobjov, Jane Idavain, Merika Rätsep, Hanna Tolonen, Mari Mäkinen, Mika Gissler, Jennifer Zeitlin, Marianne Philibert, Laure Carcaillon-Bentata, Romana Haneef, Tatjana Makovski, Martin Thißen, Stefanie Seeling, Angela Fehr, Thomas Ziese, Christina Georgakopoulou, Elena Petelos, Christog Lionis, Dimitra Lingri, Tóth Kornél, Ágnes Töll, Peter Bezzegh, István Csizmadia, Róbert Láng, Kiss Csaba, Alan Cahill, Michael Courtney, Pauline White, Kelly Ailish, Patricia Clarke, Sharon Kappala, Breda Smyth, Luigi Palmieri, Brigid Unim, Andrea Faragalli, Janis Misins, Irisa Zile, Ausra Zelviene, Audronè Astrauskiené, Guy Weber, Dorita Buttigieg, Neville Calleja, Oleg Lozan, Rodica Gramme, Mariken Tijhuis, Daniela Moye Holz, Henk Hilderink, Linda Berger-Symons, Marit de Vries, Håkon Haaheim, Frode Forland, Zuzana Nordeng, Tricia Larose, Malgorzata Strozyk, Pawel Maryniak, Krystyna Drogon, Karolina WęGrzyn, Tomasz Wisniewski, Kinga Paciorek, Paulo Nogueira, Leonor Bacelar-Nicolau, Rodrigo Feteira Santos, Luís Lapão, Mariana Peyroteo, Marília Silva Paulo, Teresa Montez, Carlos Dias, Veronica Gomez, Lucinda Oliveira das Neves, Andre Peralta-Santos, Petru Sandu, Elena Gabriela Gaftonie, Edit Fekete, Lacramioara Brinduse, Silviu Radulescu, Maja Krstic, Aleksandar Medaveric, Jan Cap, Metka Zaletel, Matej Vinko, Tatjana Kofol Bric, Inmaculada León Gómez, Carmen Rodriguez-Blazquez, M João Forjaz, Marta Marin, Amparo Larrauri, Rebeca Ramis, Asuncion Diaz, Ester Angulo-Pueyo, Cesar Garriga, Teresa Valero, Francisco Estupiñán, Sandra Garcia-Armesto, Enrique Bernal-Delgado, Juan González García, Javier Gómez-Arrue Azpiazu, Ramon Launa Garces, Teresa López-Cuadrado, Ramón Launag, Carlos Telleria, Meriam Seral, Ester Angulo-Pueyo, Lovisa Syden, Ashley Akbari, Ronan Lyons, Sarag Aldridge

**Affiliations:** Department of Public Health and Welfare, Finnish Institute for Health and Welfare (THL), Helsinki, Finland; Health Information, Sciensano, Belgium; Department of Cardiovascular, Endocrine-metabolic Diseases and Aging, Istituto Superiore di Sanità, Rome, Italy; Department of Cardiovascular, Endocrine-metabolic Diseases and Aging, Istituto Superiore di Sanità, Rome, Italy; Health Information, Sciensano, Belgium; UNIDEMI, Universidade Nova de Lisboa, Lisbon, Portugal; UNIDEMI, Universidade Nova de Lisboa, Lisbon, Portugal; International Affairs and Consultancy Department, Austrian National Public Health Institute (GÖG), Vienna, Austria; Health Information, Sciensano, Belgium

## Abstract

**Background:**

Timely and high-quality population-level health information is needed to support evidence-informed decision-making, for planning and evaluation of prevention, care and cure activities as well as for research to generate new knowledge. FAIR (Findable, Accessible, Interoperable and Reusable) principles are one of the key elements supporting health research and making it more cost-effective through the reuse of already existing data. Currently, health data are in many countries dispersed and difficult to find and access.

**Methods:**

Two EU Public Health Programmes co-funded Joint Actions, Information for Action (InfAct) and Population Health Information Research Infrastructure (PHIRI) have established a European Health Information Portal, a web-based service, to facilitate better findability, access, interoperability and reuse of existing health information.

**Results:**

The European Health Information Portal (www.healthinformationportal.eu) has been established including sections on National Nodes, data sources, publications, health information projects within countries and across Europe, research networks and research infrastructures, ethical and legal issues for health information exchange and use, capacity-building activities in all areas of population health and a dedicated COVID-19 section.

**Conclusions:**

The European Health Information Portal, being a central place for a wide range of population health information from EU Member States, is an information source for researchers, policy-makers and other relevant stakeholders. It is important to ensure the sustainability of the portal, especially in light of the European Health Data Space (EHDS) Regulation proposal and its requirements regarding the secondary use of health data.

## Introduction

Health information is often seen as data or other information about the health of an individual and healthcare services. This is usually related to a person’s medical history, i.e. patient data that also include an aggregated level information on healthcare use. The concept of health information can be extended to population health information which would cover the health outcomes and determinants more broadly than just patient records.^1^ There are several ways to obtain population health information such as self-reported questionnaires, or record linkage of various data sources.^2^

Accurate and up-to-date health information is essential not only to healthcare providers but also needed by decision-makers to support their evidence-informed decision-making, for researchers to generate new evidence, and for other stakeholders in their actions to support population health. Health information is also needed for health education and training. The Health in All Policies emphasizes that health is relevant not only for the health sector but also for all sectors, such as education, environment, finance and technology, and therefore an intersectoral approach would be needed.^3^ It is important to understand that interlinkage between health and other sectors is bidirectional, health for all policies.^4^

During the past 3 years, the coronavirus disease 2019 (COVID-19) pandemic has clearly demonstrated the need for accurate and timely health information and has helped different actors improve the use of health information, make this information easily findable and accessible and facilitate sharing between countries and research groups within and across countries.^5^^,^^6^ At the same time, fast demographic changes are experienced by most of the European countries which include rapidly ageing populations, urbanization and widening social inequalities leading to increasing burden of diseases and multimorbidity, persistent health inequalities and growing pressure on health systems. This, together with current economic turmoil requires real-time, high-quality and reliable health information to support researchers and decision-makers in finding evidence and tools to tackle these health challenges.

Numerous actors generate and disseminate health information at regional, national and international levels. Regionally and nationally, statistical offices, public health institutes, healthcare providers, etc. generate health information at both patient and population levels. International organizations like the EU through Eurostat, the European Centre for Disease Control (ECDC) and the World Health Organization (WHO), and the Organization for Economic Co-operation and Development (OECD), collaborate with individual countries to process and distribute health information. Additional to these mandatory national data collections, research projects generate valuable population-level health information.^7^ All this information is dispersed around different websites and portals, making it challenging to gain a comprehensive overview and access it in a user-friendly manner.

To respond to this problem of fragmented information, several initiatives have been made to centralize information or to establish metadata catalogues. Some of these catalogues exist for life sciences, like the European Research Infrastructure for Biobanks (BBMRI-ERIC) sample/data locator^8^ and ELIXIR Data Platform.^9^ Until now, none of these existing catalogues or portals were focused on population health.

The Joint Action on Health Information (InfAct)^10^ and the Population Health Information Research Infrastructure (PHIRI)^11^ have been working to establish a one-stop shop for health information in Europe. The aim is to fill the gap in Europe’s health information landscape by concentrating on population health information. PHIRI was developed to facilitate and generate the best available evidence for researching the health and well-being of populations impacted by COVID-19.^11^^,^^12^

## Methods

The concept and development of the Health Information Portal (HIP)^12^ started during the InfAct Joint Action in 2018–21. The status of availability of health information, including health and healthcare service-related data, research networks^7^ and projects, health reporting^13^ and capacity-building activities^14^ in Europe was reviewed. The review demonstrated severe and worrisome fragmentation of the information resulting in the recommendation to establish a European health information infrastructure^15^ where the HIP would be one of the key pillars provided to strengthen evidence-informed policymaking and research. Other pillars were training and capacity building and direct exchange of expertise and good practices.

During the InfAct Joint Action, a conceptual framework for the HIP was prepared including a structure for the presentation of information in a standardized format. One of the key concepts developed during the InfAct Joint Action was National Nodes. Health information is dispersed over several institutes in most of the European countries, without regular communication among these bodies. This easily leads to limited interoperability of available information and in some cases inhibits the exchange of data resulting in a loss of research opportunities and support for decision-making. Therefore, a clear need for more coordinated national actions was identified and National Nodes were seen as a possible approach to tackle this. National Nodes are defined as organizational entities within each country, that should bring together different national actors and stakeholders collecting, providing, and using health information. These actors and stakeholders include, for example, the National Statistical Office, the national public health institutes and representatives from the ministries of health, research and/or science and social insurance institutes.

The work on the HIP started during the InfAct Project, when InfAct Steering Committee defined the first structure of the HIP. Key experts in health information across Europe were consulted and the sustainability of the portal was always kept in mind. It was designed in such a way that any project could contribute to it and the content would stay relevant. Also, the focus was given to the user and what a user would want to find on the portal.

The technical setting up and development of the HIP has been mainly done during PHIRI, even though some initial pages were established already during the InfAct Project. The portal has been set up using the open-source web content management system DRUPAL.^16^

For each section of the HIP, a standardized metadata template was prepared to ensure structured data entry and easy searchability of information on the portal. The entire portal is built to support the FAIR (findability, accessibility, interoperability and reusability) principles.^17^ Metadata descriptions are documented and published and are updated when modifications are done, or new modules are added.^18^ Metadata descriptions are based on common metadata standards: Data Catalogue Vocabulary (DCAT)^19^ for health information sources and Data Documentation Initiative^20^ for other materials such as publications and project descriptions. To enhance the findability and machine readability of information on the HIP, web pages also include Schema.org^21^ metadata information.

Information about the HIP is obtained through (i) the National Nodes, (ii) desk research and (iii) regular exchange meetings conducted during PHIRI. National Nodes have been in a key role in providing information on national health and healthcare data sources as well as national health information projects and publications and as facilitators for the COVID-19 section of the portal.

### Health Information Portal

**Figure 1:**
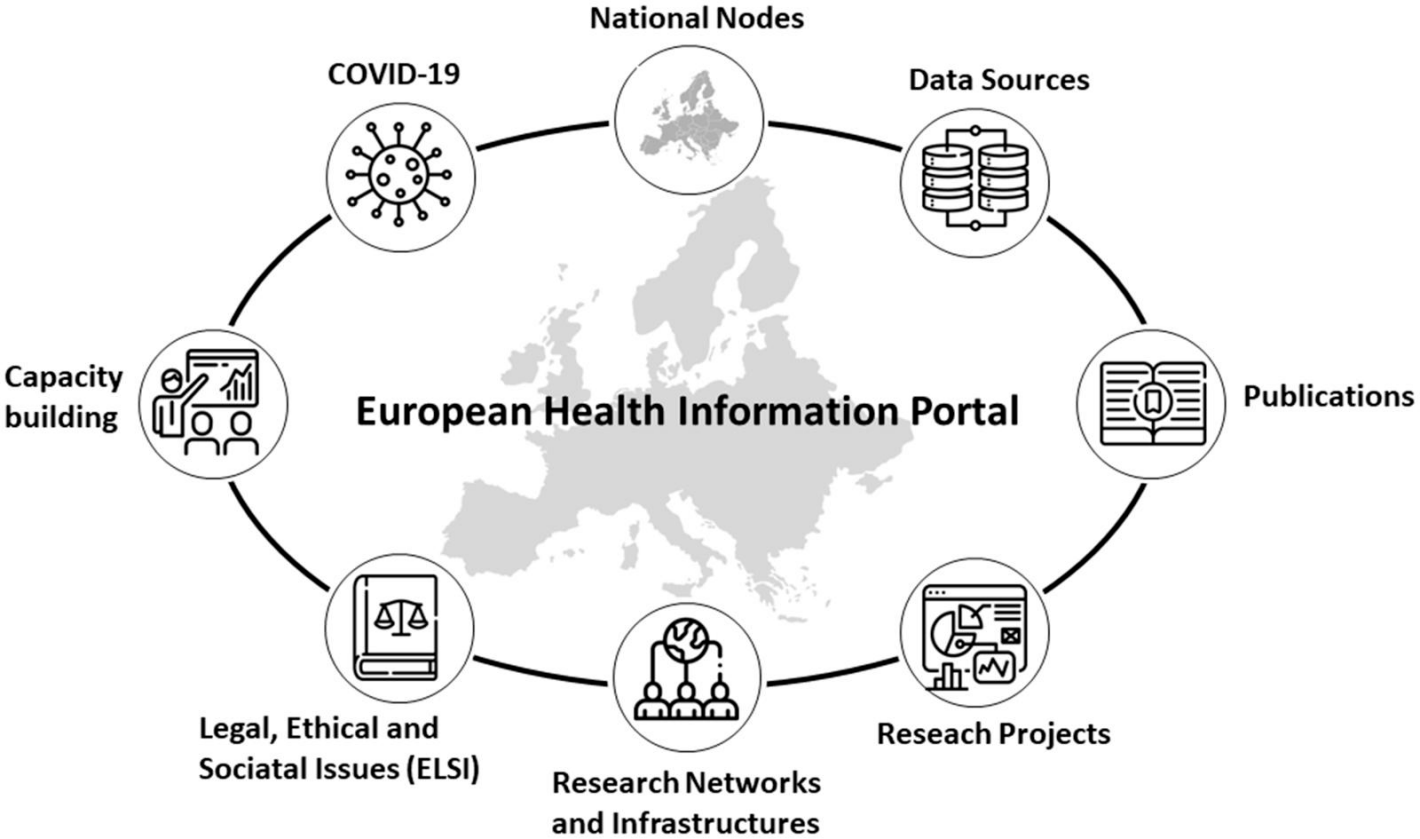
Sections of the European Health Information Portal

The HIP (https://www.healthinformationportal.eu/) provides information in different sections, both as a list of all items on the portal and through a devoted search function. The sections include National Nodes, data sources, publications, health information projects within countries and across Europe, research networks and research infrastructures, ethical and legal issues for health information exchange and use, capacity-building activities in all areas of population health and a COVID-19 section (figure 1).

### National Nodes

Up to June 2023, the section on National Nodes included information on 27 countries (EU Members States except Denmark, Luxembourg and Greece, and Albania, Bosnia and Hertzegovina, Norway, UK). For each country, a description of health information systems is provided. This includes information about main institutes that collect health information in the country and their responsibilities, and national data protection regulations. Through specific National Nodes pages, information on data sources, publications, projects and involvement in European-level research networks of this specific country can be accessed.

### Data sources

The section on data sources provides a metadata catalogue of population health data in European countries. Currently, this catalogue includes over 300 data sources in different categories: survey/interview data (e.g. health examination/interview surveys), administrative data (e.g. hospital discharge data), population data (e.g. causes of death), registry data (e.g. cancer registries), outpatient utilization data (e.g. morbidity data), social health insurance data, surveillance data of infectious diseases, biobank/sample/specimen data (e.g. biobanks), calculations (e.g. cancer statistics, life expectancy), hospital resources and healthcare administrative area resources (e.g. register of entities performing medicinal activities), hospital resources and healthcare resources (e.g. register of pharmacies), hospitalization statistics of the portals of the national health system (e.g. hospital waiting lists), customer record data (e.g. food consumption data), observational study data (e.g. cohort studies), multiple sources and other records.

### Publications

The publication section will not duplicate information already findable through existing publication catalogues such as PubMed, Medline, Web of Science, etc. The focus is on non-indexed publications, so-called ‘grey literature’, such as national reports, policy briefs, National HIPs/websites and guidelines. These are often publications which are difficult to find through a web search or organization websites due to language barriers, or publications which are not open access, but may include important and interesting information for researchers and stakeholders.

### National- and European-level projects on health information

The section of National- and European-level projects on health information has been built through two approaches. First, National Nodes have provided information about their national health information-related projects. Additional to those national projects, international databases have been searched to identify population-based COVID-19-related projects to be listed in the portal. This search has excluded COVID-19-related clinical trials^22^ as well as surveillance systems^23^ since those are already listed elsewhere.

### Research networks and research infrastructures

The section on research networks and infrastructures includes information on some of the established health information-related networks such as the European Injury Database, and research infrastructures such as ELIXIR^9^ and BBMRI-ERIC.^8^

### Ethical and legal issues for health information exchange and use

The section on ethical and legal issues for health information exchange and use includes existing EU-level legislation, and international and EU-level ethical guidelines related to health data collection, use and exchange. It also has examples of best practices obtained through the literature search and survey conducted among PHIRI partners.

### Capacity-building activities on health information

The section on capacity-building activities includes information about available capacity-building activities on COVID-19, and population health and health information more broadly. There are two sections on capacity building: one on activities identified through a desk search and one on the European School on Health Information. A catalogue of capacity-building activities identified through a desk search include different types of training activities such as courses, e-learning materials, massive open online courses, podcasts, seminars/webinars and symposiums. The European School on Health Information was established during the InfAct Project with the 1st edition organized in 2019^24^^,^^25^ and the 2nd edition during PHIRI in 2023. During the PHIRI project, a series of training activities are also organized to contribute to the European School on Health Information, and all these are listed on the portal together with links to the training materials.

### COVID-19 section of the portal

The section on COVID-19 includes two components: one about the COVID-19 Rapid Exchange Forum (REF) and the other one addressing COVID-19-related policy measures. The REF was established to respond to the urgent need for a quick exchange of COVID-19-related information among national actors in an informal arena to support decision-making and research. The outcomes of these bi-weekly REF meetings are documented on the HIP to summarize discussions and country actions for posed questions. Subjects ranged from discussions on the benefits of face shields versus nose-mouth masks, how to handle testing in tourist areas or how to deal with long COVID to topics in connection to the Health Security Committee, e.g. on how many doses of vaccine countries plan to order. Since Q4/2022, the topics in the REF have been expanded to further population health topics, e.g. the state of digital death certificates in EU/EEA.

The second section offers a dashboard on COVID-19 policy measures that contains short summaries of key policy measures for COVID-19 mitigation including lockdown, travel and mobility restrictions, impact on the educational system, test, trace and isolate activities, personal protection measures and other topics such as vaccination strategies. The section features an interactive map, and it is possible to search by country or by measure. During the pandemic phase of COVID-19, the content complemented the country-wise concise overview pages prepared by ECDC and Joint Research Centre (JRC), offering details on the situation in the countries.

### Other sections

The HIP can also foster other sections highlighting the outcomes of different projects. Currently, for example, the portal has a section from PHIRI on Federated demonstrators presenting key outputs from PHIRI methodology for federated data analysis through four case studies. The portal also has a section about COVID-19 health information system assessments conducted during PHIRI.

### Sustainability and future development

**Figure 2:**
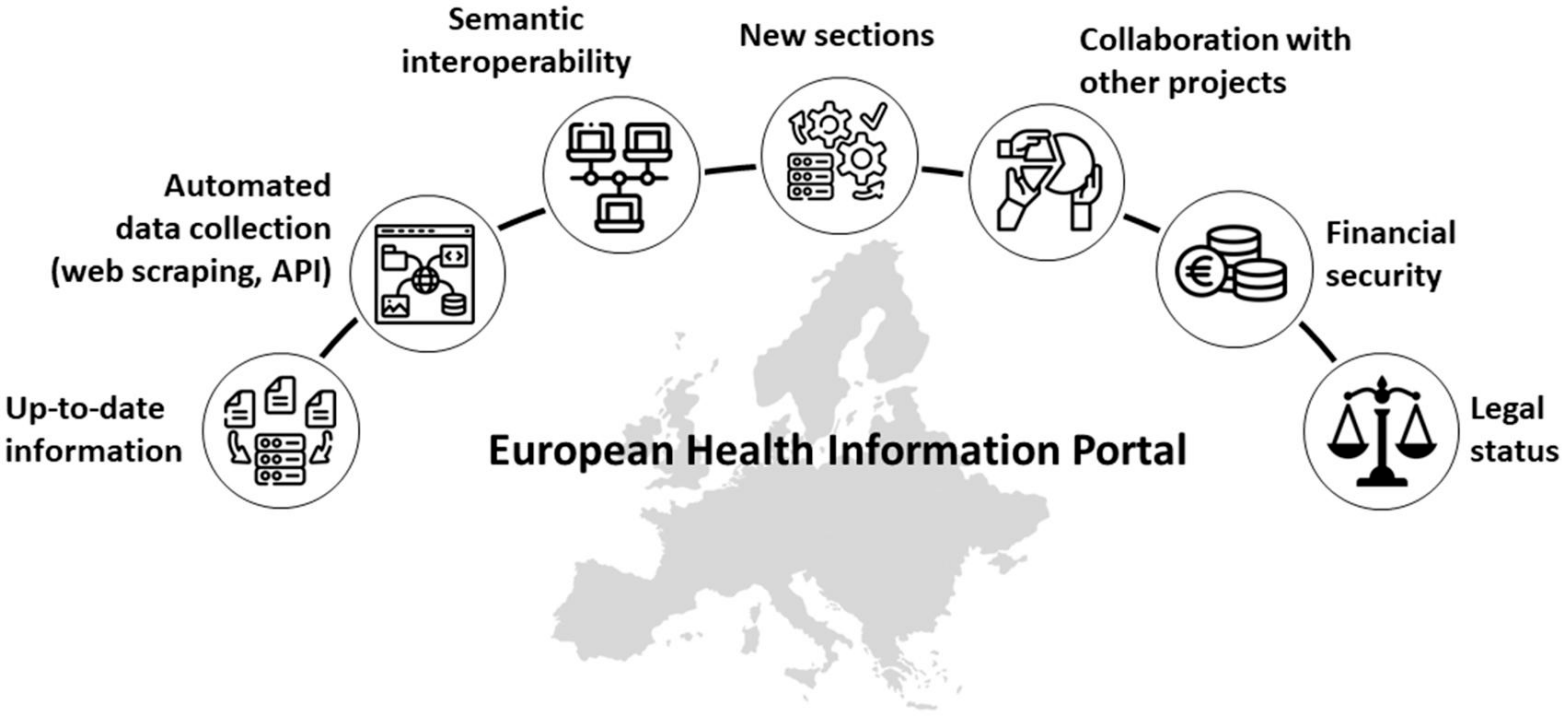
Required components for the sustainability of the European Health Information Portal

For the sustainability of the European HIP, it is essential to have resources and processes to maintain and develop the HIP (figure 2). Updated information on all sections is needed, but also, further development of the portal by adding new sections and features is important to keep up with the information needs of rapidly changing societies and future population health challenges. To make maintenance of the portal and updating of the information as cost-effective as possible, manual work for data and information collection and entry should be minimized. Making use of international standards allows metadata harvesting from different repositories that use the same standards and have an Application Programming Interface (API) in place.

There is also a need for increasing semantic interoperability of the portal by mapping the conceptual framework to popular ontologies to increase machine readability of the information. It is also important to ensure extensive use of Schema.org metadata descriptions on the portal to increase the findability of information by search engines such as Google.

In the first phase, the HIP focused mostly on COVID-19-related information due to the urgent need for this specific information at the time. The HIP is designed to include a wide range of health information, not only COVID-19 related. Different sections of HIP already cover different aspects of health information, and this will be extended in the future. This is strongly supported by the development of the European Health Data Space (EHDS) Regulation,^26^ which determines the legal framework for health data sharing and use in the EU, for both primary and secondary use. The EHDS requires that countries establish a national data catalogue to provide a metadata for their health data, adhering to the standards of the European Commission, such as DCAT. Since the HIP is also using the DCAT standard for the metadata description of health data sources, this allows both the transfer of material from HIP to a national data catalogue and vice versa through the API. As such, the HIP is designed to be able to already accommodate the needs of the EHDS regulation and support countries which do not have a metadata catalogue to use the HIP for theirs.

In the EHDS regulation proposal,^26^ there is also a part regarding the secondary use of health data and health data access bodies. The National Nodes established during the InfAct and PHIRI Projects are ideal candidates to take over this role in some countries.

The PHIRI consortium collaborates with several other EU-funded projects (e.g. HealthyCloud,^27^ BY-COVID,^28^ HealthData@EUpilot^29^ and ELIXIR^9^) and international organizations (e.g. ECDC) on the use of data catalogue established on HIP. This collaboration is important not only to avoid unnecessary duplication of work and information but also to enhance the use of the portal by a broader audience and in other health domains. The HIP can be extended to serve the needs of other ongoing or new projects, providing them with a landing page and collecting activities on health information in one portal.

If the HIP is used by several different projects to share their outcomes, this also increases the possibility for future funding of the portal maintenance and development. Obviously, in the long-term, project-based funding is not a sustainable solution. Therefore, other solutions such as the possibility of applying for the European Strategic Forum on Research Infrastructures roadmap and through that obtaining an ERIC status for the HIP or establishing a non-profit organization to support the HIP are in progress.

## Conclusions

European HIP, being a central place for a wide range of population health information from EU Member States, provides a one-in-a-kind information source for researchers, decision-makers and other relevant stakeholders. Currently, there are on average over 800 visits per month to the portal and this is expected to increase when awareness about HIP increases. Knowledge of available health information sources allows researchers to find already existing data, making research more cost-effective, but also fosters possibilities for innovative research.

For decision-makers and other relevant stakeholders, information on HIP provides basis to identify the knowledge gaps and supports evidence-informed decision-making as well as planning of well-targeted prevention activities.

In the future, it will be increasingly important to combine health information with information from other sectors such as environment, transport, technology, agriculture and economy to obtain a more holistic understanding of the impact of different determinants on health but also how health affects different sectors. For this, having a common set of, European-level standards for data governance and sharing are needed.

## Data Availability

No new data were generated or analyzed in support of this research.

## Disclaimer

The content of this manuscript represents the views of the authors only and is their sole responsibility. The European Research Executive Agency (REA) and the European Commission are not responsible for any use that may be made of the information in contain.

*Conflicts of interest*: None declared.
Key points

- Representative and timely data are needed to support evidence-informed decision-making.
- The European Health Information Portal (HIP) is a central place for a wide range of population health information from EU Member States.
- FAIR (Findable, Accessible, Interoperable and Reusable) principles are key elements of the HIP.

## References

[1] Kindig D , Stoddart G. What is population health? Am J Public Health 2003;93:380–3. 12604476 10.2105/ajph.93.3.380 PMC1447747

[2] Unim B , Mattei E, Carle F, et al Health data collection methods and procedures across EU member states: finding from the InfAct Joint Action on health information. Arch Public Health 2022;80:17. 34986889 10.1186/s13690-021-00780-4 PMC8728985

[3] Leppo K , Ollila E, Peña S, et al Health in All Policies—Seizing Opportunities, Implementing Policies. Finland: Ministry of Social Affairs and Health, 2013. http://urn.fi/URN:ISBN:978-952-00-3407-8 (22 August 2023, date last accessed).

[4] Greer SL , Falkenbach M, Siciliani L, et al From health in all policies to health for all policies. Lancet 2022;7:e732–20. 10.1016/S2468-2667(22)00155-4 PMC9330081 35907422

[5] Gray R. Coronavirus accelerates drive to share health data across borders. *Horizon. The EU Reseach & Innovation Magazine* 2020. https://ec.europa.eu/research-and-innovation/en/horizon-magazine/coronavirus-accelerates-drive-share-health-data-across-borders (22 August 2023, date last accessed).

[6] Dron L , Kalatharan V, Gupta A, et al Data capture and sharing in the COVID-19 pandemic: a cause of concern. Langet Digital Health 2022;4:E748–e756. 10.1016/S2589-7500(22)00147-9 PMC9489064 36150783

[7] Unim B , Haverinen E, Mattei E, et al Mapping European research networks providing health data: results from the InfAct Joint Action on health information. Arch Public Health 2022;80:23. 35012667 10.1186/s13690-021-00766-2 PMC8744039

[8] BBMRI-ERIC. *Sample/Data Locator*. https://www.bbmri-eric.eu/services/sample-locator/ (22 August 2023, date last accessed).

[9] ELIXIR. *Data Platform*. https://elixir-europe.org/platforms/data (22 August 2023, date last accessed).

[10] Joint Action on Health Information (InfAct). https://www.inf-act.eu/ (22 August 2023, date last accessed).

[11] Population Health Information Research Infrastructure (PHIRI). https://www.phiri.eu/ (22 August 2023, date last accessed).

[12] European Health Information Portal. 10.25504/FAIRsharing.8690f1 https://www.healthinformationportal.eu (22 August 2023, date last accessed).

[13] Thissen M , Seeling S, Achterberg P, et al Overview of national health reporting in the EU and quality criteria for public health reports—results of the Joint Action InfAct. Arch Public Health 2021;79:229. 34933687 10.1186/s13690-021-00753-7 PMC8692080

[14] Lapão L , Beja A, Fernandes GV, et al *Report on Mapping Needs, Capacities and Training Programmes in Health Information*. https://www.inf-act.eu/sites/inf-act.eu/files/2020-01/InfAct_D6.1_MappingCapacitiesAndTrainingProgrammes.pdf (22 August 2023, date last accessed).

[15] Sarmiento-Suárez R , Padron-Monedero A, Bogaert P, et al The InfAct proposal for a sustainable European health information infrastructure on population health: the Distributed Infrastructure on Population Health (DIPoH). Rch Public Health 2022; 80:139. 10.1186/s13690-022-00844-z PMC9113621 35581661

[16] Drupal. https://www.drupal.org (22 August 2023, date last accessed).

[17] GoFair. *FAIR Principles*. https://www.go-fair.org/fair-principles/ (22 August 2023, date last accessed).

[18] Tolonen H , Mäkinen M, Bogaert P, et al *Documentation and User Guide for the Health Information Portal. Metadata* Description. Zenodo. 2022. 10.5281/zenodo.6413408 (22 August 2023, date last accessed).

[19] W3C. *Data Catalog Vocabulary (DCAT)—Version 2*. https://www.w3.org/TR/vocab-dcat-2/ (22 August 2023, date last accessed).

[20] DDI. Document. *Discover and Interoperate*. https://ddialliance.org/ (22 August 2023, date last accessed).

[21] Schema.org. https://schema.org/ (22 August 2023, date last accessed).

[22] U.S. National Library of Medicine. *Views of COVID-19 Studies Listed on ClinicalTrials.gov (Beta)*. https://clinicaltrials.gov/ct2/covid_view (22 August 2023, date last accessed).

[23] ECDC. *The European Surveillance System (TESSy)*. https://www.ecdc.europa.eu/en/publications-data/european-surveillance-system-tessy (22 August 2023, date last accessed).

[24] Lapão L , Beja A, Tolonen H, et al *INFACT—Sustainable Capacity Building Programme (European Health Information Training Programme—EHITP)*. https://www.inf-act.eu/sites/inf-act.eu/files/2020-04/EHITP%20-%20INFACT%20D6.2.pdf (22 August 2023, date last accessed).

[25] European Health Information Training Programme 1st European School on Health Information. Final Report. https://www.inf-act.eu/sites/inf-act.eu/files/2021-05/INFACT_Task%206.3-Report_%20FINAL_MARCH_0.pdf (22 August 2023, date last accessed).

[26] EUR-Lex. Proposal for a REGULATION OF THE EUROPEAN PARLIAMENT AND OF THE COUNCIL on the European Health Data Space. https://eur-lex.europa.eu/legal-content/EN/TXT/?uri=CELEX%3A52022PC0197 (22 August 2023, date last accessed).

[27] Health Research & Innovation Cloud (HealthyCloud). https://healthycloud.eu/ (22 August 2023, date last accessed).

[28] BY-COVID. https://by-covid.org/ (22 August 2023, date last accessed).

[29] EHDS. *Health Data@EU Pilot*. https://www.ehds2pilot.eu/ (22 August 2023, date last accessed).

